# *NAMPT* and *NAPRT1*: novel polymorphisms and distribution of variants between normal tissues and tumor samples

**DOI:** 10.1038/srep06311

**Published:** 2014-09-09

**Authors:** Sara Duarte-Pereira, Sarah S. Silva, Luísa Azevedo, Luísa Castro, António Amorim, Raquel M. Silva

**Affiliations:** 1 IPATIMUP - Institute of Molecular Pathology and Immunology of the University of Porto, Rua Dr. Roberto Frias s/n, 4200-465 Porto, Portugal; 2 Faculty of Sciences, University of Porto, Rua do Campo Alegre, 4169-007 Porto, Portugal; 3 IEETA - Institute of Electronics and Telematics Engineering of Aveiro, University of Aveiro, Santiago Campus, 3810-193 Aveiro, Portugal

## Abstract

Nicotinamide phosphoribosyltransferase (NAMPT) and nicotinate phosphoribosyltransferase domain containing 1 (NAPRT1) are the main human NAD salvage enzymes. NAD regulates energy metabolism and cell signaling, and the enzymes that control NAD availability are linked to pathologies such as cancer and neurodegeneration. Here, we have screened normal and tumor samples from different tissues and populations of origin for mutations in human *NAMPT* and *NAPRT1*, and evaluated their potential pathogenicity. We have identified several novel polymorphisms and showed that *NAPRT1* has a greater genetic diversity than *NAMPT*, where any alteration can have a greater functional impact. Some variants presented different frequencies between normal and tumor samples that were most likely related to their population of origin. The novel mutations described that affect protein structure or expression levels can be functionally relevant and should be considered in a disease context. Particularly, mutations that decrease NAPRT1 expression can predict the usefulness of Nicotinic Acid in tumor treatments with NAMPT inhibitors.

Nicotinamide phosphoribosyltransferase (NAMPT) and nicotinate phosphoribosyltransferase domain containing 1 (NAPRT1) are major enzymes in the cellular metabolism. Their substrates, nicotinamide (Nam) and nicotinic acid (NA), respectively, are important precursors in Nicotinamide Adenine Dinucleotide (NAD) biosynthesis^1^. Several pathways contribute to the replenishment of the NAD pool and, in mammalian cells, Nam is the predominant precursor^2^,^3^ while NA is more effective in increasing NAD levels in some tissues^4^. Given that NAD participates as a coenzyme in oxidation-reduction reactions, but is also a substrate of NAD-consuming enzymes that are involved in gene expression regulation, DNA repair or cell death, it is expected that NAD availability directly influence pathological conditions^5^,^6^. Increasing evidence points to a role of NAD salvage enzymes in cancer and neurodegeneration^5^,^6^,^7^,^8^.

Originally, NAMPT was identified as pre-B-cell colony enhancing factor 1 (PBEF1)^9^ and as visfatin^10^, and its role in NAD biosynthesis was recognized later^11^. NAMPT functions in immunity, metabolism, and stress responses in physiology and pathophysiology^12^. As energy generation and NAD–dependent signaling are crucial for cell proliferation and cancer progression, NAMPT inhibitors have emerged as promising antitumor drugs^5^,^13^,^14^.

NAPRT1 increases intracellular NAD levels and prevents oxidative stress^4^. Since NA increases NAD levels via NAPRT1 action, the oral administration of NA was suggested to ameliorate NAD depletion conditions, namely, as cytoprotective agent in cancer treatments with NAMPT inhibitors^13^,^14^,^15^.

Genotype-phenotype associations in a disease context raise interest in single nucleotide polymorphisms (SNPs) detection. SNPs in human *NAMPT* associated with pathological conditions such as glucose and lipid metabolism alterations^16^,^17^, acute lung injury^18^, coronary artery disease^19^ and type 2 diabetes^20^, are located in non-coding regions. Polymorphisms in *NAMPT* promoter are related to plasma insulin levels^21^ and increased cholesterol^22^. For *NAPRT1* the only association with disease, namely, attention deficit hyperactivity disorder^23^, is the synonymous SNP rs2290416 (G428, exon 10), responsible for differences in protein expression^24^.

Motivated by the importance of these enzymes in metabolism and homeostasis, and their involvement in several human diseases, we present the results of large-scale analyses that involved more than 200 samples from both normal and tumor origin in order to characterize potentially relevant mutations. The impact of novel variants at the structural and/or functional levels is discussed.

## Results

### *NAMPT* and *NAPRT1* gene polymorphisms

Despite all the available literature on *NAMPT* polymorphisms, public databases show that *NAMPT* is less diverse than *NAPRT1*. Yet, little is known about the impact of *NAPRT1* polymorphisms. We analyzed 96 DNA samples from a control population, focusing in mutation hotspots of the *NAMPT* and *NAPRT1* genes (Supplementary Table S1). At the beginning of this study, the regions analyzed were chosen based on the information retrieved from public databases and the literature. For *NAMPT*, we found four intronic variants with minor allele frequency (MAF) lower than 0.05 (Table 1). One frequent SNP was detected in exon 7 (rs2302559, g.21735A>G), corresponding to Ser301. The frequencies observed in our samples were consistent with results from the 1000 Genomes Project (Table 1).

**Figure 1:**
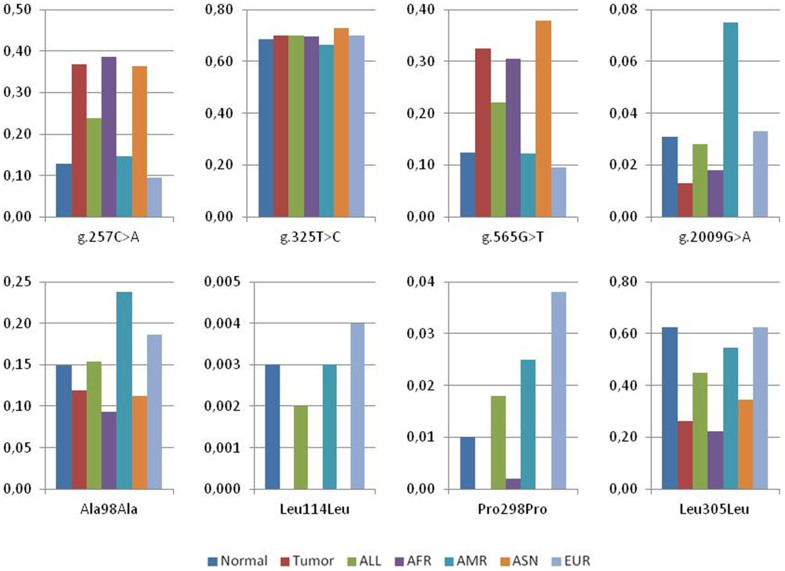
Allele frequencies of NAPRT1 genetic variants. The graphs show the frequencies calculated from our results, both in normal and in tumor samples, for four non-coding and four silent variants. Data from 1000 Genomes reflecting the frequencies in different populations is also included. The novel polymorphisms g.2013A>G and g.676C>G as well as the two missense mutations Val106Met and Arg332Cys were not included since no data was available in the Ensembl Genome Browser. ALL: all individuals from phase 1 of the 1000 Genomes Project; AFR: African; AMR: American; ASN: East Asian; EUR: European.

The above mentioned 96 samples and additional 53 normal tissue samples, consisting in blood, stomach and colon from different ethnic groups, were analyzed for mutations in *NAPRT1* (Table 2 and Figure 1). Among the five silent substitutions detected, one at Val142 codon (g.676C>G) remains unreported. The synonymous variant g.1803C>T at Leu305 (rs872935) is the most frequent and found in nearly 63% of the alleles, consistent with 1000 Genomes data for the Caucasian population. Two missense variants (rs200364051, p.Val106Met and rs35975875, p.Arg332Cys) were detected with frequencies of 0.5% and 0.3%, respectively, and four non-coding variants were also identified. From the eleven *NAPRT1* variants found, five had MAF<0.05, including two missense, two silent and one non-coding, in accordance to 1000 Genomes data (Table 2). Sequencing the entire *NAPRT1* gene allowed the discovery of five additional non-coding variants, plus one novel deletion in intron 8 (g.2542_2544delCCC) (Table 2).

To evaluate whether the variants have different prevalence in pathological conditions and normal tissues, 80 DNA samples from colon and gastric tumors were also analyzed. Apart from two silent alterations, which were also detected in normal samples (g.468C>T and g.1803C>T, at Ala98 and Leu305, respectively), no missense mutations were found in *NAPRT1*. Curiously, the g.1803C>T variant was more frequent in the normal population than in tumor samples (63% and 26%, respectively). From the five intronic variants found, g.257C>A and g.565G>T were more frequent in tumor than in normal samples (around 30% and 13%, respectively) (Table 2 and Figure 1). In the 1000 Genomes data, these are also more frequent in the Asiatic population, consistent with the fact that the tumor samples analyzed were from Asian origin, while control samples were mostly Caucasian. Thus, these results may be explained by different frequencies between populations rather than differences observed between control and tumor samples. In addition, we identified a polymorphism which was not previously described in intron 7 (g.2013A>G). The remaining variants showed similar frequencies between populations (Table 2 and Figure 1). To validate the population effect on the allelic differences, we used re-sampling statistics^25^,^26^ and compared *NAPRT1* bootstrap confidence intervals (95%) for mean allele frequencies found in our data (control and tumor samples) with the 1000 Genomes allele frequencies (European and Asian populations). For most variants, we observed a similar trend between normal and European population frequencies, as well as between tumor and Asian population frequencies (Supplementary Table S3).

### Impact of missense variants in the protein structures

**Figure 2:**
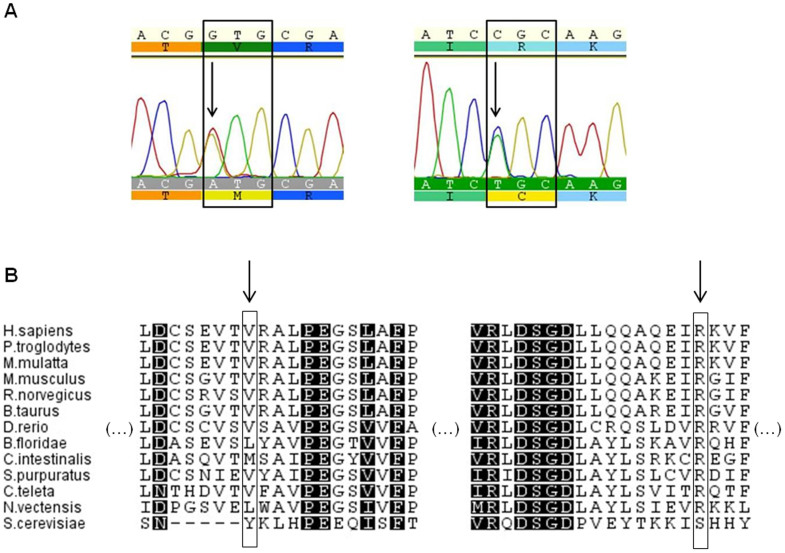
Missense mutations found in human NAPRT1 gene. (A). Electropherograms showing 341C>T (left) and 1019C>T (right) replacements in heterozygosity that lead to Val106Met and Arg332Cys substitutions, respectively. Geneious software was used to visualize the sequences. (B). Localization of the amino acids in a multi-species alignment. V106 (left arrow) is conserved in vertebrates, and is substituted by interchangeable residues (Val/Leu) in invertebrate species. R332 (right arrow) is highly conserved, except in the yeast protein.

Since there is no human NAPRT1 structure available, models of the protein were predicted by homology modeling and by *ab initio* modeling, based on *Saccharomyces cerevisae*^27^ and *Enterococcus faecalis* NAPRTases. After assessing quality (Supplementary Figure S3) the model with the lowest z-score was chosen (Supplementary Figure S4) to evaluate the structural impact of the NAPRT1 missense mutations found in our samples (Figure 2A).

**Figure 3:**
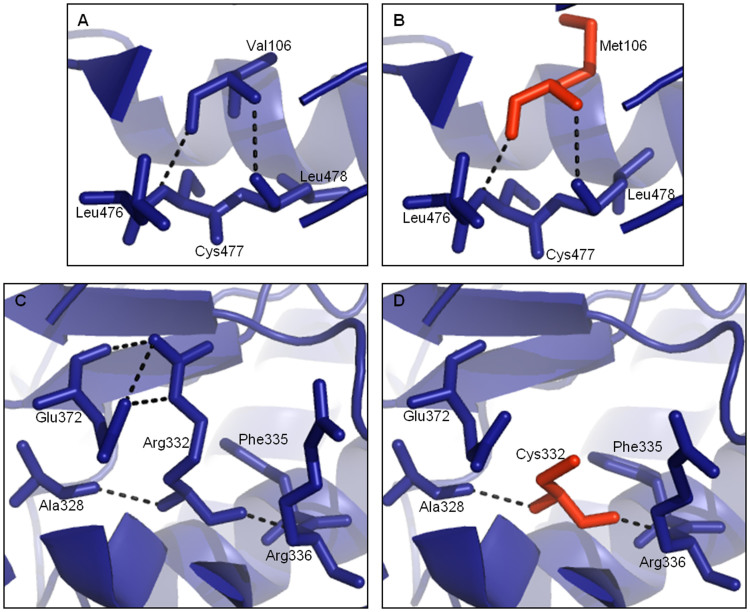
Structural impact of the human NAPRT1 missense mutations. Val106 (A) interacts with Leu476, Cys477 and Leu478 and in the model with Met106 (B – red) the same interactions are maintained. Arg332 (C) establishes contacts with Ala328, Arg336, Phe335 and Glu372. Cys332 (D – red) loses polar contact with Glu372.

Taking into consideration residue conservation, Arg332 is invariant throughout all Metazoan species analyzed whereas Val106 is substituted by interchangeable residues (valine and leucine, except for yeast), and even methionine in *Ciona intestinalis* (Figure 2B). Accordingly, the SIFT^28^ and PolyPhen^29^ predictions considered the Val106Met substitution tolerated/benign and the Arg332Cys mutation as deleterious/probably damaging, respectively (Supplementary Table S4). In the structural models, the Val106Met replacement does not cause an apparent modification in the protein structure (Figure 3A and B) however, in the Arg332Cys replacement a distinct network of H-bond contacts is observed when a cysteine residue replaces the ancestral arginine (Figure 3C and D). Specifically, Arg332 establishes polar contacts with Ala328, Arg336, Phe335 and Glu372, and the polar contact with Glu372 is lost with the Cys332.

**Figure 4:**
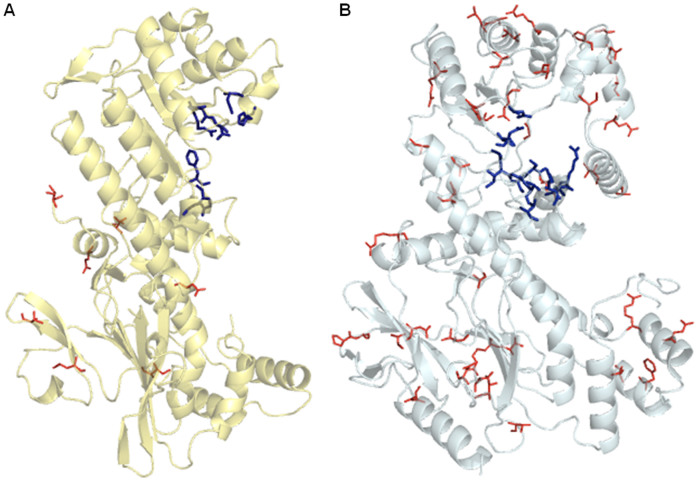
NAMPT and NAPRT1 missense spectra. (A). The PDB structure of human NAMPT (PDB id: 3DKJ) representing only one of the two chains of the dimeric molecule. The active site residues (blue sticks) are Phe193, Arg311, Asp313, Asp279, His247; from chain 2 (not represented) the residues involved are Tyr18, Lys415, Lys423^30^. The missense mutations are shown in red lines. (B). The structure of the human NAPRT1 was predicted with I-TASSER (http://zhanglab.ccmb.med.umich.edu/I-TASSER/) and represents one chain of the predicted dimer. The blue sticks represent the active sites as predicted by The Conserved Domain Database (CDD:29617), from NCBI^43^. The red lines illustrate the missense mutation residue location, which are all over the molecule.

Using the NAMPT structure^30^ and our model for NAPRT1, we located all the missense mutations described so far as well as the predicted active sites in both proteins (Figure 4). Missense mutations found in NAMPT are far from the active sites or the dimer interface (Figure 4A), thus, their potential impact on the enzyme structure and/or function should be limited. On the other hand, NAPRT1 missense mutations, which are abundant, are spread all over the molecule (Figure 4B). This is consistent with SIFT^28^and PolyPhen^29^ predictions as well (Supplementary Table S4).

## Discussion

NAMPT and NAPRT1 are important enzymes in NAD metabolism, both in normal and in pathological conditions. In this study, we targeted the regions where most missense mutations were located. Variation data from Ensembl Genome Browser (Dec2010 - Jan2013) indicated an increasing number of coding region alterations in both genes, although more evident for *NAPRT1* (Supplementary Figure S5). For instance, in the same period, 40 missense and 20 silent new mutations were described in *NAPRT1*, compared to 8 and 15, respectively, in *NAMPT*. This shows that *NAPRT1* is more permissive to mutations and, thus, much more polymorphic than *NAMPT*, in which a strong purifying selection must be acting on.

Our results confirmed that *NAMPT* has a lower genetic diversity than *NAPRT1*. We found a smaller number of alterations and observed that missense mutations are rare in the human gene. In fact, we detected only intronic and synonymous variants. Intronic SNPs in *NAMPT* are associated with disease phenotypes related to glucose and lipid metabolism and other metabolic and vascular traits (see^31^ and^32^ for review), possibly due to alterations in splicing, protein binding or methylation sites that will affect the levels of protein expression. As for synonymous mutations, it has been recently shown that rs2302559 correlates with *NAMPT* serum levels^33^, thus, the impact of these novel variants is yet to be determined.

In *NAPRT1* we detected two missense and five silent variants in samples from healthy individuals. One of these, g.676C>G, is an unreported alteration in exon 3 (Val142Val). Although commonly considered silent, synonymous mutations can have a phenotypic effect and be implicated in human disease^26^,^33^,^34^. Protein expression or conformation can be affected by speeding up or slowing down protein synthesis or by affecting splicing.

Using a novel methodology to estimate the pathogenicity of human genetic variants^35^, we observed that many *NAPRT1* variants here described are associated with splicing sites (Supplementary Table S5). Given the location of the Val142 near the exon border, we used the NetGene2 online server, which predicts human splice sites^36^, to evaluate the impact of this new polymorphism. We observed that an alternative splice site near the mutated g.676C>G is predicted with higher confidence than the splice site that determines the exon 3-intron 3 boundary in the reference sequence, resulting in an alternatively spliced transcript. As a recent study suggests that synonymous mutations change the sequences that regulate splicing in oncogenes, and are frequently associated with cancer^26^, the impact of this, and other, synonymous mutations should be considered in further studies.

The absence of NAPRT1 expression has been reported in different types of cancer^14^,^37^, thus, we expected to find a higher number of alterations in *NAPRT1* that could affect protein expression. This is of particular importance to validate NA as a chemoprotectant in the treatment of cancer patients with NAMPT inhibitors, which would be effective in NAPRT1-negative tumors only^38^. Curiously, no missense mutations were detected in the tumor samples studied, further supporting the role of intronic and synonymous mutations in promoting changes in protein expression, as discussed above. Recent work also shows that epistasis influences *NAPRT1* gene expression^39^, suggesting that further studies will be necessary to establish which SNP or SNP associations are preponderant in defining NAPRT1-negative phenotypes.

Although the human NAMPT enzyme is well characterized, with known active site and dimer interface residues^30^,^40^,^41^, for human NAPRT1 no protein structure is yet available. A recent study^42^ on the kinetic characterization of this enzyme identified residues involved in activity, and used the *Thermoplasma acidophilum* NAPRTase to infer structural information. To study the structural impact of missense variants, we also built models for human NAPRT1, and evaluated Val106Met and Arg332Cys mutations considering residue conservation and changes in bond contacts. We further located known missense mutations of NAPRT1 and NAMPT in their respective structures, as well as residues involved in the active center^30^,^43^. For NAMPT, none of the missense mutations would interfere directly with the active center of the enzyme or with the dimer interface, whereas in NAPRT1, the missense mutations are located all over the molecule. Despite no direct bond to active center residues was detected, these mutations may influence assembly of the active dimer form, resulting in a dysfunctional or inactive protein.

The growing number of mutations described in *NAPRT1* that occur all over the molecule is somewhat puzzling. Although it could be secondary in tissues that express *NAMPT*, *NAPRT1* is useful for cells that lack *NAMPT*, such as neuron, and for cells in the digestive tract due to abundance of NA from bacterial metabolism^44^. Moreover, as NA is a more efficient NAD precursor^4^, situations with high NAD demand or *NAMPT* impairment would benefit from the activity of *NAPRT1*. Further studies are required to elucidate if the variants found in this study are correlated with protein expression levels, not only in physiological normal conditions but also in a disease context.

## Methods

### Samples

All samples used in this study are commercially available and were acquired as extracted DNA. Donors have given written, informed consent for their samples to be used for research purposes. DNA samples (108 healthy UK Caucasian blood donors) of the ECACC (European Collection of Cell Cultures) Human Random Control DNA Panel were purchased from Sigma-Aldrich (St. Louis, MO, USA). Human Adult Genomic DNA panels were obtained from AMS Biotechnology (Abigdon, U.K.), and included DNA from blood (25 samples, from 15 Caucasian, 2 Asian, 3 African American, 1 Center/S American, 3 Hispanic and 1 N/A individuals), colon and stomach tissues (16 samples, from 6 Caucasian and 10 Asian individuals) and colon and gastric tumors (80 samples, all of Asian origin).

### Polymerase chain reactions

Polymerase chain reactions (PCR) were prepared using HotStarTaq® Master Mix Kit (Qiagen, Germantown, MD, USA), 0.2 µM (final concentration) of each primer and 10% Q solution (Qiagen). Primer sequences to *NAMPT* and *NAPRT1* fragments, containing relevant mutation sites described in public databases (NCBI, Ensembl) and the literature, are provided in Supplementary Table S1.

PCR amplification was as follows: 95°C (15 min), 35 cycles at 94°C (30 sec), 58–64°C (1 min) and 72°C (1 min), and final extension at 72°C (10 min). Amplification products were separated by horizontal polyacrylamide gel electrophoresis and visualized by silver staining.

### Sequencing analysis

PCR products were purified with ExoSAP-IT (USB Corporation, Santa Clara, CA, USA) according to the manufacturer's instructions and sequenced with the ABI Big Dye Terminator Cycle Sequencing Ready Reaction kit v3.1 (Applied Biosystems, Life Technologies Corporation, Carlsbad, CA, USA). Fragments were analyzed in an ABI PRISM 3130xl (Applied Biosystems). Sequences were aligned using Geneious v.5.5 (created by Biomatters, available from http://www.geneious.com/).

### Protein alignments

Human NAMPT (NP_005737) and NAPRT1 (NP_660202) amino acid sequences were aligned with homologue proteins. Sequences were retrieved from the NCBI (http://www.ncbi.nlm.nih.gov/) and Joint Genome Institute (JGI) Genome Portal (genome.jgi.doe.gov/) databases (Supplementary Table S2). Alignments were performed using MUSCLE^45^ incorporated in Geneious v.5.5 and are shown in Supplementary Figures S1 and S2.

### Homology modeling and structure visualization

The human NAPRT1 protein sequence was used as template to search for the best E-value PDB using the NCBI BlastP analysis^46^ This search identified structures from *Enterococcus faecalis,* *Thermoplasma acidophilum* and *Saccharomyces cerevisiae* as optimal structural templates. *E. faecalis* putative nicotinate phosphoribosyltransferase and *S. cerevisiae* Npt1p structures (PDB id: 2F7F and 1VLP) were used as templates in MODELLER^47^ to build human NAPRT1 structural models by homology modeling. Additionally, the I-TASSER online server^48^,^49^ was used to predict NAPRT1 structure by *ab initio* modeling. Accuracy of the models (Supplementary Figure S3) was estimated using ProSA-web (https://prosa.services.came.sbg.ac.at/prosa.php), as previously described^50^,^51^. Models of the NAPRT1 variants Val106Met and Arg332Cys were built in MODELLER, using as template the I-TASSER predicted structure (Supplementary Figure S4). The human NAMPT structure used corresponds to the PDB id: 3DKJ^30^. All structures were visualized in Pymol v1.1r1^52^.

### Mutation analyses

Re-sampling statistics were performed by bootstrap analysis using MATLAB (R2011a, MathWorks, Natick, MA, USA). The method used for the construction of the bootstrap confidence intervals (95%) was the accelerated bias-correction (Supplementary Table S3).

Pathogenicity prediction of the variants was performed as follows. Available information of the SIFT^28^ and PolyPhen^29^ predictions, for all dbSNP missense mutations in the human *NAMPT* and *NAPRT1* reference transcripts (ENST00000222553 and ENST00000449291), were retrieved from Ensembl^53^ (release 75 - February 2014) (Supplementary Table S4).

For all the variants found in our study, the pathogenicity was also scored according to CADD (http://cadd.gs.washington.edu/score)^35^. Variants were retrieved from the 1000 Genomes Browser^54^ (http://browser.1000genomes.org/index.html) as vcf files, corresponding to the coordinates of *NAMPT* and *NAPRT1* genes (chr7:105888731-105926772 and chr8:144656955-144660819). Variants were selected according to the position on the chromosome and scores are shown in Supplementary Table S5.

## Author Contributions

R.M.S. designed and supervised the study. S.D.P. and S.S.S. performed the experiments. L.A., L.C., A.A. and R.M.S. analyzed the data. S.D.P. and R.M.S. wrote the paper. All authors revised and approved the manuscript.

## Supplementary Material

Supplementary Information SUPPLEMENTARY INFO

## Figures and Tables

**Table 1:** Allele frequencies of human *NAMPT* genetic variants. Four intronic and one silent variants were found in human *NAMPT* from healthy donors (n = 96). The frequencies from 1000 Genomes were retrieved from the Ensembl Genome Browser

Ref ID	Variant type	Exon or intron	Nucleotide change	Amino acid change	Frequencies n = 96	*1000 Genomes*
rs41430346	Non-coding	intron 3	g.12484G>C	-	0.979/0.021	0.971/0.029
rs375379216	Non-coding	intron 3	g.12498C>T	-	0.995/0.005	-
rs112487390	Non-coding	intron 4	g.12774C>G	-	0.989/0.011	0.958/0.042
rs2302559	Silent	exon 7	g.21735A>G	Ser301Ser	0.375/0.625	0.335/0.665
rs144888107	Non-coding	intron 7	g.21850C>G	-	0.979/0.021	0.994/0.006

**Table 2:** Allele frequencies of human *NAPRT1* genetic variants. Five intronic, five silent and two missense variants were found in normal (n = 149) and tumor (n = 80) samples, including two novel polymorphic sites. Five additional intronic variants were found in the complete sequence of five samples (n.d., frequencies not determined), plus a previously undescribed deletion. The frequencies from 1000 Genomes were retrieved from the Ensembl Genome Browser

Ref ID	Variant type	Exon or intron	Nucleotide change	Amino acid change	Normal n = 149	Tumor n = 80	*1000 Genomes*
rs2015562	Non-coding	intron 1	g.257C>A	-	0.872/0.128	0.631/0.369	0.761/0.239
rs896953	Non-coding	intron 1	g.325T>C	-	0.316/0.684	0.300/0.700	0.299/0.701
rs896954	Silent	exon 2	g.468C>T	Ala98Ala	0.851/0.149	0.881/0.119	0.846/0.154
rs200364051	Missense	exon 2	g.490G>A	Val106Met	0.995/0.005	-	n.a.
rs145565666	Silent	exon 2	g.516C>A	Leu114Leu	0.997/0.003	-	0.998/0.002
rs2305496	Non-coding	intron 2	g.565G>T	-	0.875/0.125	0.675/0.325	0.779/0.221
-	Silent	exon 3	g.676C>G	Val142Val	0.915/0.085	-	-
rs12678314	Non-coding	intron 3	g.906T>C	-	n.d.	n.d.	0.272/0.728
rs744650	Silent	exon 7	g.1784C>T	Pro298Pro	0.990/0.010	-	0.982/0.018
rs872935	Silent	exon 7	g.1803C>T	Leu305Leu	0.375/0.625	0.738/0.263	0.551/0.449
rs35975875	Missense	exon 7	g.1884C>T	Arg332Cys	0.997/0.003	-	0.997/0.003
rs114291348	Non-coding	intron 7	g.2009G>A	-	0.969/0.031	0.987/0.013	0.972/0.028
-	Non-coding	intron 7	g.2013A>G	-	-	0.994/0.006	-
rs896955	Non-coding	intron 7	g.2144G>A	-	n.d.	n.d.	0.763/0.237
-	Non-coding	intron 8	g.2542_2544 delCCC	-	n.d.	n.d.	-
rs2290417	Non-coding	intron 11	g.3245C>G	-	n.d.	n.d.	0.315/0.685
rs77951814	Non-coding	intron 12	g.3362G>C	-	n.d.	n.d.	0.913/0.087
