# Supplementary material for: NAMPT and NAPRT1: novel polymorphisms and distribution of variants between normal tissues and tumor samples
Source: Sci Rep. 2014 Sep 9;4:6311. doi: 10.1038/srep06311 (PMC4158320; doi:10.1038/srep06311)
Supplement: Supplementary Information — SUPPLEMENTARY INFO [file srep06311-s1.pdf]

NAMPT and *NAPRT1*: novel polymorphisms and distribution of variants between normal tissues and tumor samples.

Sara Duarte-Pereira[a], Sarah S. Silva[a], Luísa Azevedo[a,b], Luísa Castro[c], António Amorim[a,b] and Raquel M. Silva[a*]

[a]IPATIMUP - Institute of Molecular Pathology and Immunology of the University of Porto, Rua Dr. Roberto Frias s/n, 4200-465 Porto, Portugal;
[b]Faculty of Sciences, University of Porto, Rua do Campo Alegre, 4169-007 Porto, Portugal
[c]IEETA - Institute of Electronics and Telematics Engineering of Aveiro, University of Aveiro, Santiago Campus, 3810-193 Aveiro, Portugal

*Corresponding author:
Raquel M. Silva
Present address:
IEETA, University of Aveiro, Santiago Campus, 3810-193 Aveiro, Portugal
Email: raquelsilva@ua.pt
Tel: + 351 234 370 500
Fax: + 351 234 370 545

SUPPLEMENTARY FIGURES

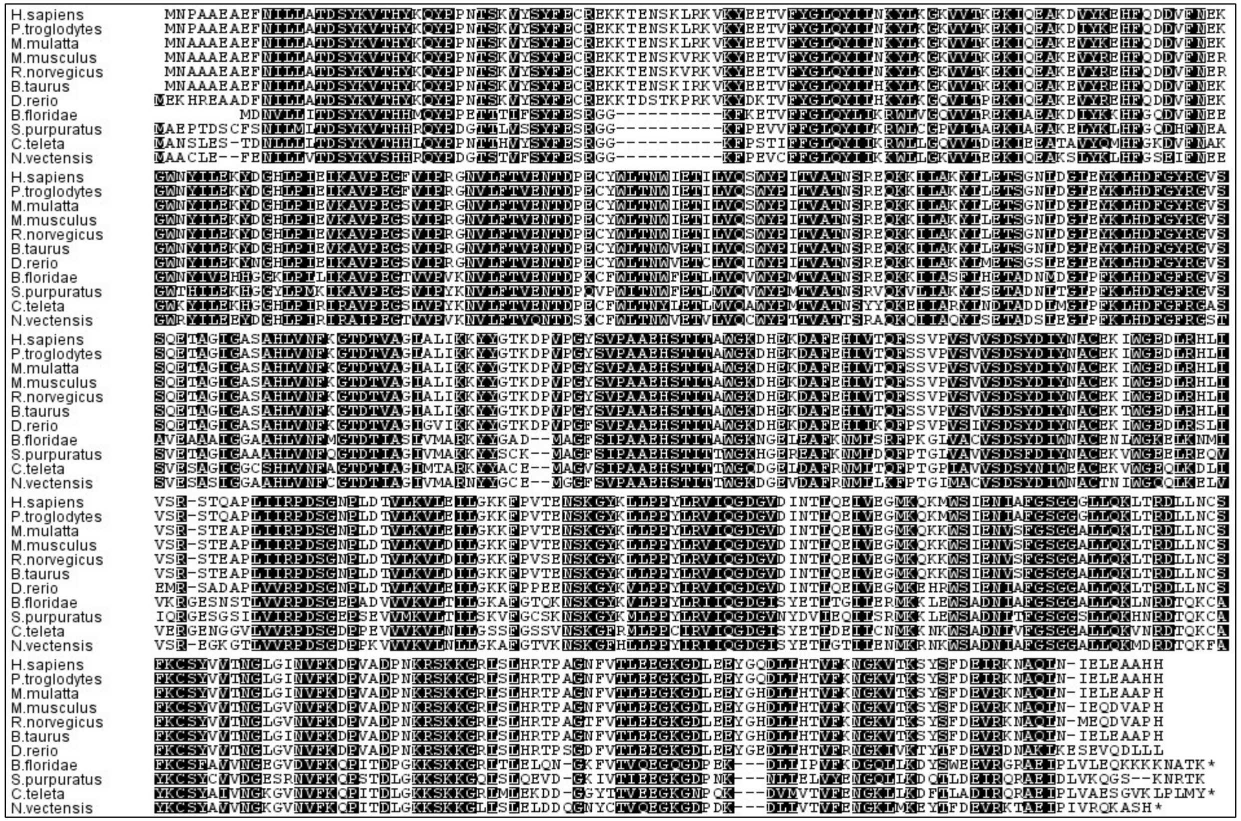

Supplementary Fig. S1. Amino acid sequences alignment of NAMPT in different species.

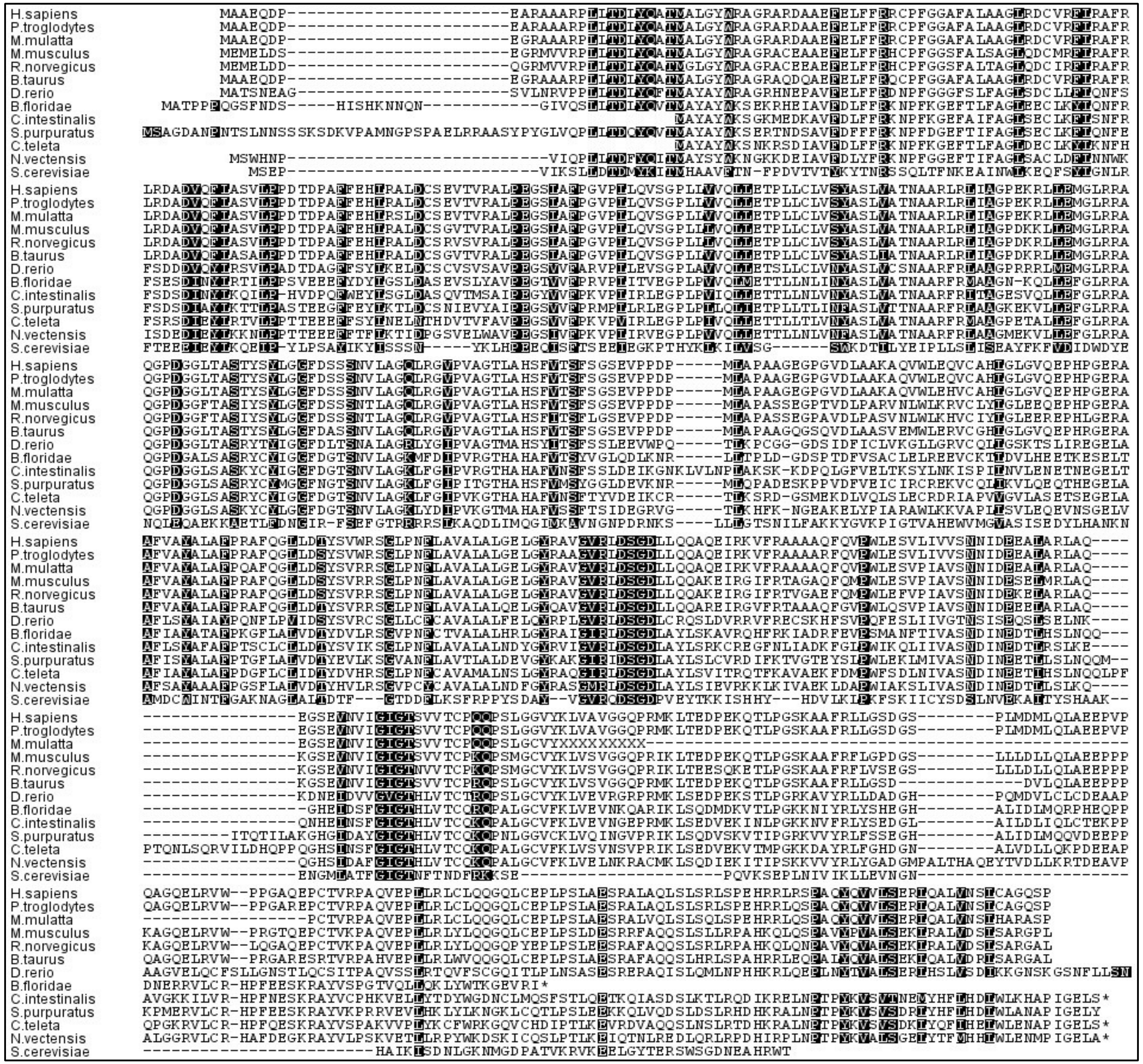

Supplementary Fig. S2. Amino acid sequences alignment of NAPRT1 in different species.

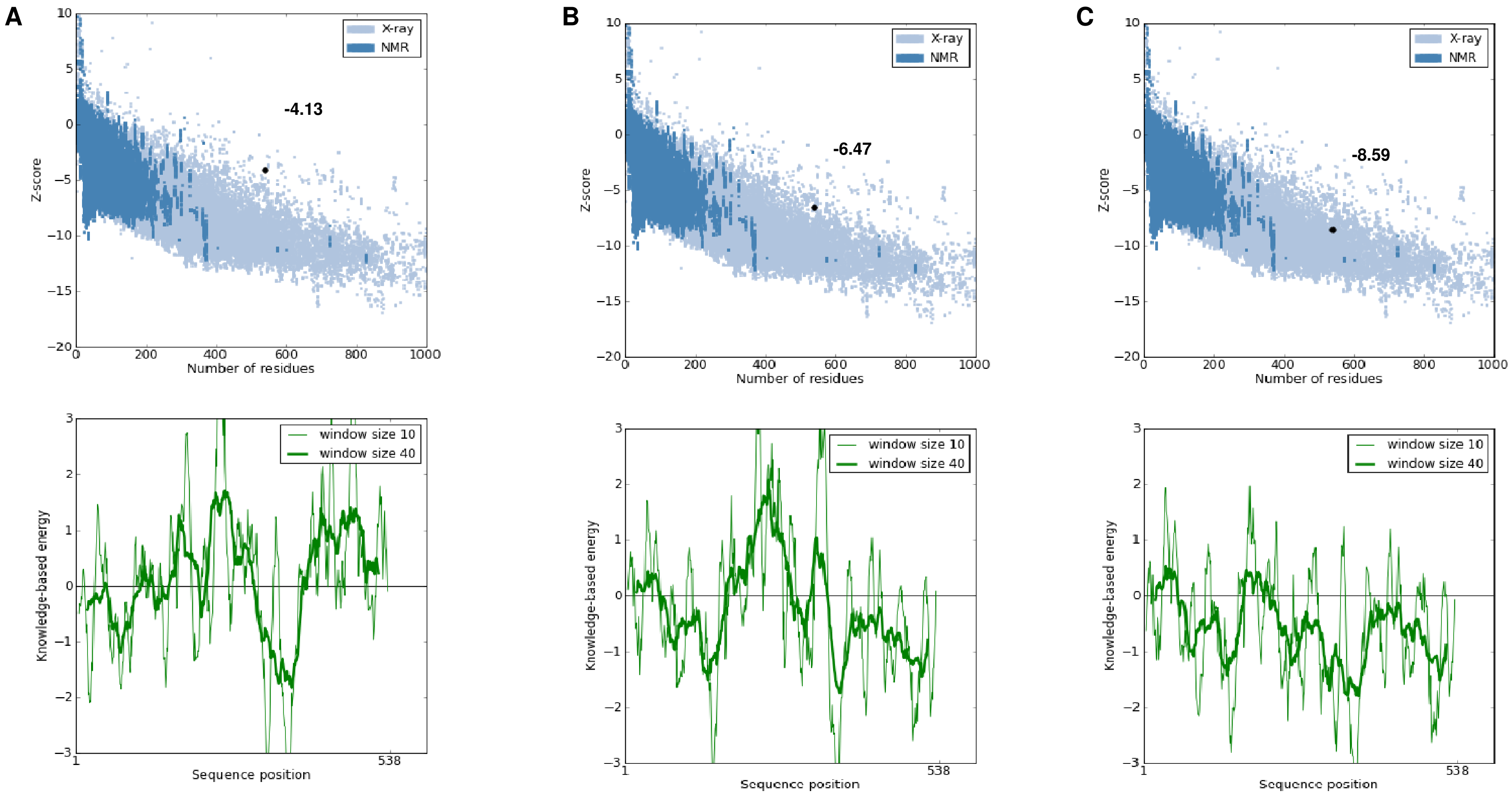

Supplementary Fig. S3. Quality evaluation of the modeled structures of the human NAPRT1 protein.
Evaluation of model quality for the predicted 3D structures of human NAPRT1, both overall (top) and locally (bottom) as estimated in ProSA-web. The models were predicted by MODELLER based on the *S. cerevisae* Npt1 (**A**), the *E. faecalis* putative NAPRT1 protein (**B**), or predicted by I-TASSER (**C**), and the best z-score value was obtained in the latter.

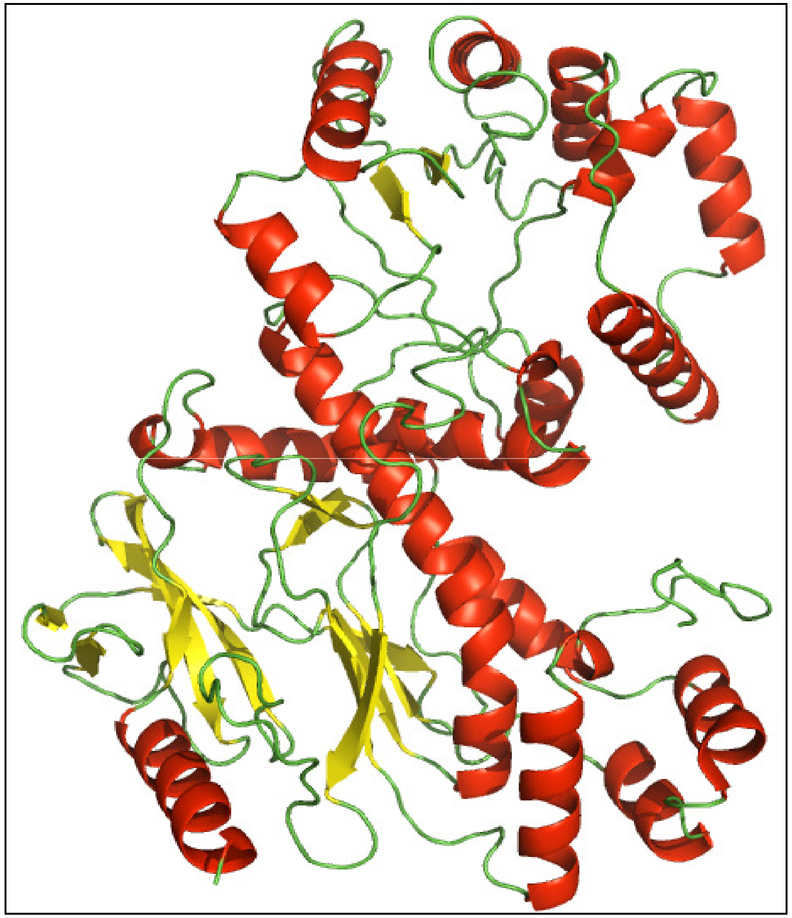

Supplementary Fig. S4. Predicted tertiary structure of human NAPRT1.
The model was obtained using the I-TASSER server. α-Helices are in red, β-sheets are in yellow and loops are in green.

A

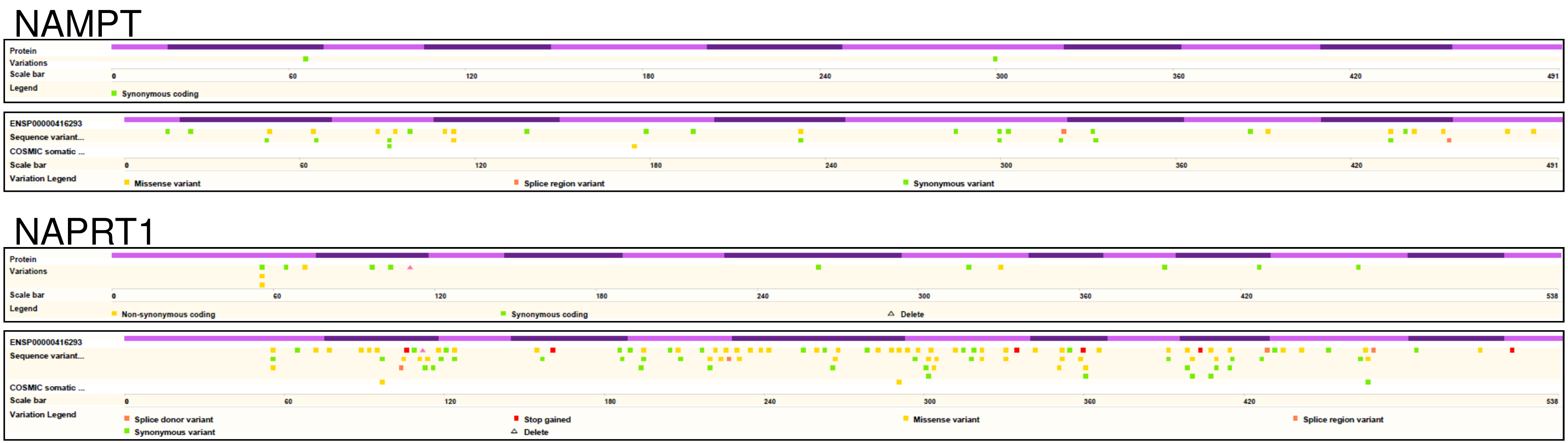

B

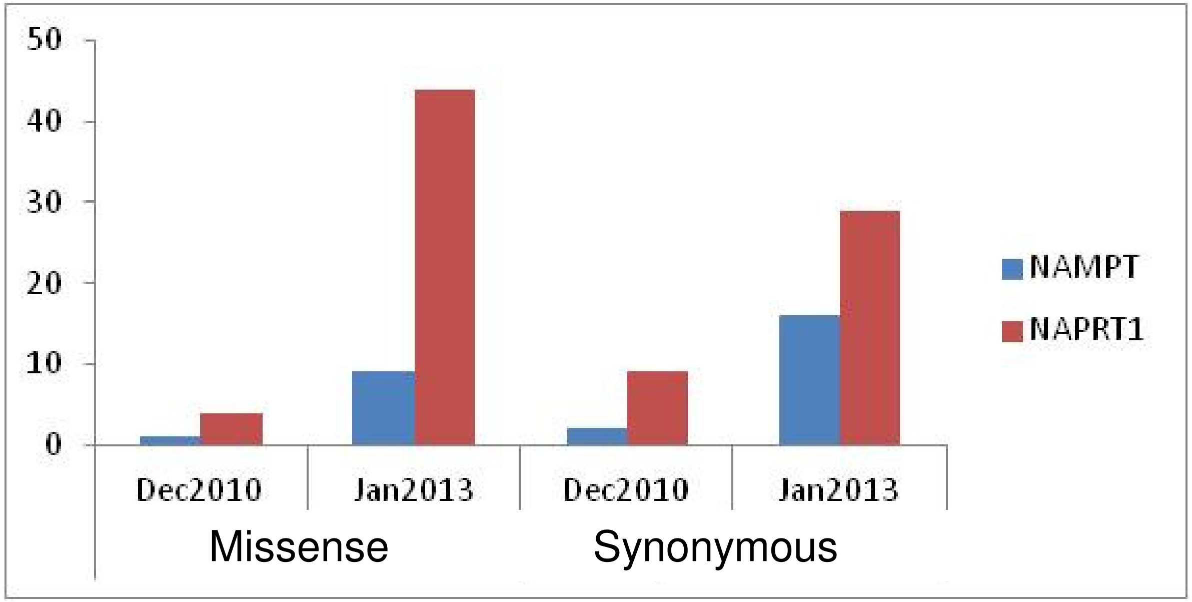

Supplementary Fig. S5. NAMPT and NAPRT1 variation data retrieved from Ensembl Genome Browser.
A. Comparison between 2010 and 2013 shows the increasing number of mutations reported for both genes.
B. Missense and synonymous variants underline that *NAPRT1* has a higher genetic diversity than *NAMPT*.

Supplementary Tables

Supplementary Table S1. Oligonucleotide sequences used for amplification of NAMPT and NAPRT1 genes.

Gene	Location	Primer forward (5'-3')	Primer reverse (5'-3')	Annealing temperature (ºC)	Product size (bp)
NAMPT	Intron 1 - Intron 2	CTTCAAGCCTTTTCTGTTGTG	CTTAGAACATCAAACACACACC	60	424
	Intron 3 - Intron 4	AGCACGTGGCAGCATTAAAC	GATGAGGAAATTGAAGCCTGG	58	502
	Exon 7 - Intron 7	CATAACAGCTTGGGGGAAAG	CTCTCTCTGGGCTGCAACT	58	362
	Intron 8 -Intron 9	CAGTCTTGGATGCTTCATTC	CTCCCTTCTTTCCTTGTTTCTG	58	402
NAPRT1	Exon 1 - Intron 1	ACCTCTACCAGGCCACCATG	GGTAACTGCGCTGAGACCAG	64	310
	Intron 1 - Intron 3	CTGGTCTCAGCGCAGTTACC	CAGCCCACCTGGCGTAGC	60	425
	Exon 6 - Intron 7	CTACAGCGTGTGGAGGTGAG	CACCTGTGCTCACCTTCTGC	60	404

Supplementary Table S2. Sequences of NAMPT and NAPRT1 protein orthologues.
The table lists the species and the associated protein identifier and source used in the protein alignment.

	NAMPT			NAPRT1		
**Species**	**ID**	**source**	**size (aa)**	**ID**	**source**	**size (aa)**
Homo sapiens	NP_005737	NCBI	491	NP_660202	NCBI	538
Pan troglodytes	JAA31706	NCBI	491	JAA00578	NCBI	538
Macaca mulatta	AFI35917	NCBI	491	XP_001097478.2	NCBI	481
Mus musculus	NP_067499	NCBI	491	NP_766195	NCBI	538
Rattus norvegicus	NP_808789	NCBI	491	NP_997492	NCBI	538
Bos taurus	NP_001231070	NCBI	491	NP_001092327	NCBI	533
Danio rerio	CU928083.1	Ensembl	493	NP_957184	NCBI	548
Branchiostoma floridae	288618	JGI	475	278419	JGI	497
Ciona intestinalis	-	-	-	211042	JGI	524
Strongylocentrotus purpuratus	XP_782393	NCBI	480	XP_787773.3	NCBI	584
Capitella teleta	162451	JGI	482	175488	JGI	540
Nematostella vectensis	135670	JGI	476	89511	JGI	547
Saccharomyces cerevisiae	-	-	-	NP_014852	NCBI	429

Suplementary Table S3. Bootstrap analyses between allele frequencies of the *NAPRT1* variants obtained in our study versus allele frequencies from the 1000 Genomes Project. The method used for the construction of the bootstrap confidence intervals (95%) was the accelerated bias-correction available in MATLAB (R2011a, MathWorks, Natick, MA, USA).

		Bootstrap CI		1000Genomes		InsideCIBootstrap?
		left	right			
rs2015562	Control	0,097	0,174	EUR_AF	0,09	no
	Tumor	0,294	0,444	ASN_AF	0,36	yes
rs896953	Control	0,618	0,725	EUR_AF	0,7	yes
	Tumor	0,625	0,763	ASN_AF	0,73	yes
rs896954	Control	0,107	0,191	EUR_AF	0,19	yes
	Tumor	0,075	0,181	ASN_AF	0,11	yes
rs145565666	Control	0,000	0,017	EUR_AF	0,004	yes
	Tumor	-	-	ASN_AF	n/a	-
rs2305496	Control	0,091	0,168	EUR_AF	0,09	no
	Tumor	0,263	0,406	ASN_AF	0,38	yes
rs744650	Control	0,003	0,030	EUR_AF	0,04	no
	Tumor	-	-	ASN_AF	n/a	-
rs872935	Control	0,567	0,688	EUR_AF	0,63	yes
	Tumor	0,200	0,331	ASN_AF	0,35	no
rs35975875	Control	0,000	0,023	EUR_AF	0,01	yes
	Tumor	-	-	ASN_AF	n/a	-
rs114291348	Control	0,013	0,063	EUR_AF	0,03	yes
	Tumor	0,000	0,039	ASN_AF	n/a	-

Suplementary Table S4. Predicted impact of all dbSNP missense variants for the human *NAMPT* and *NAPRT1 genes* , according to SIFT and PolyPhen calculations. Variants were retrieved from Ensembl (release 75 - February 2014), considering the reference transcripts ENST00000222553 and ENST00000449291, respectively.

Gene	ID	Chr:bp	Alleles	Global MAF	AA	AA coord	SIFT		PolyPhen	
NAMPT	rs202025145	7:1058915547:105891554	A/G	0.001(G)	I/T	484	tolerated	0,42	benign	0
	rs140819103	7:1058915817:105891581	T/G	-	E/A	475	tolerated	0,15	benign	0,005
	rs376047293	7:1058934707:105893470	T/C	-	Y/C	453	tolerated	0,17	benign	0,047
	rs138426703	7:1058935007:105893500	A/G	-	L/P	443	deleterious	0,03	benign	0,015
	rs146170636	7:1058935247:105893524	G/A	0.001(A)	T/M	435	tolerated	0,1	possibly damaging	0,764
	rs61738828	7:1058948617:105894861	A/C	-	D/E	393	deleterious	0	possibly damaging	0,874
	rs148806435	7:1059089557:105908955	G/A	-	T/M	233	tolerated	0,19	possibly damaging	0,904
	rs61746104	7:1059130827:105913082	A/G	-	I/T	114	deleterious	0,01	benign	0,108
	rs142386213	7:1059130837:105913083	T/C	-	I/V	114	tolerated	0,93	benign	0,003
	rs370987898	7:1059130917:105913091	T/C	-	H/R	111	tolerated	0,42	benign	0
	rs370874972	7:1059154297:105915429	T/C	-	D/G	94	tolerated	0,22	benign	0,002
	rs373913615	7:1059154477:105915447	T/C	-	K/R	88	tolerated	0,51	benign	0
	rs368481844	7:1059174877:105917487	G/C	-	L/V	66	deleterious	0,05	benign	0,255
	rs139282739	7:1059175317:105917531	T/C	-	K/R	51	tolerated	0,06	benign	0,104
NAPRT1	rs199590650	8:1446571828:144657182	G/A	-	R/W	510	deleterious	0	benign	0,035
	rs368170412	8:1446573848:144657384	G/A	-	R/W	475	deleterious	0	possibly damaging	0,677
	rs199833066	8:1446573988:144657398	A/C	-	V/G	470	deleterious	0	probably damaging	0,955
	rs149178575	8:1446574058:144657405	C/T	-	A/T	468	tolerated	0,28	benign	0,056
	rs201086959	8:1446574078:144657407	G/A	-	P/L	467	deleterious	0	probably damaging	0,988
	rs141565399	8:1446574808:144657480	C/G	0.000 (G)	E/Q	443	deleterious	0	probably damaging	1
	rs147123886	8:1446575008:144657500	T/A	-	D/V	436	deleterious	0	probably damaging	1
	rs147154210	8:1446576378:144657637	G/A/T	-	T/M	416	deleterious	0	possibly damaging	0,634
	rs147154210	8:1446576378:144657637	G/A/T	-	T/K	416	deleterious	0	probably damaging	0,972
	rs139521261	8:1446576538:144657653	C/T	0.001 (T)	D/N	411	deleterious	0	probably damaging	0,936
	rs144545750	8:1446576588:144657658	G/A	-	T/I	409	deleterious	0	probably damaging	0,992
	rs369842187	8:1446576798:144657679	C/T	-	G/D	402	tolerated	0,08	probably damaging	0,999
	rs148462517	8:1446576868:144657686	C/T	-	V/M	400	deleterious	0,01	probably damaging	0,982
	rs369189989	8:1446578038:144657803	C/T	-	G/S	393	deleterious	0,02	benign	0,102

rs201030598	8:1446582568:144658256	C/T	0.002 (T)	A/T	367	tolerated	0,07	benign	0,07
rs200059887	8:1446582708:144658270	G/A	0.001 (A)	A/V	362	tolerated	0,38	benign	0,009
rs369217099	8:1446582778:144658277	C/T	-	E/K	360	deleterious	0	probably damaging	0,997
rs373535032	8:1446582978:144658297	A/G	-	V/A	353	tolerated	0,43	benign	0,006
rs144202509	8:1446582988:144658298	C/T	0.001 (T)	V/I	353	tolerated	0,11	benign	0,228
rs373926829	8:1446583008:144658300	A/T	-	I/N	352	deleterious	0	probably damaging	1
rs377542827	8:1446583278:144658327	T/C	-	Q/R	343	tolerated	0,45	benign	0,046
rs184059139	8:1446586298:144658629	C/T	0.001 (T)	R/H	332	deleterious	0,02	possibly damaging	0,868
rs35975875	8:1446586308:144658630	G/A	0.003 (A)	R/C	332	deleterious	0	probably damaging	0,999
rs372036233	8:1446586378:144658637	C/A	-	Q/H	329	tolerated	0,59	benign	0,013
rs201149658	8:1446586558:144658655	G/T	0.000 (T)	D/E	323	deleterious	0	probably damaging	0,995
rs188697057	8:1446586568:144658656	T/C	0.000 (C)	D/G	323	deleterious	0	probably damaging	0,996
rs374121375	8:1446586878:144658687	G/A	-	R/W	313	deleterious	0	probably damaging	0,946
rs145806784	8:1446587078:144658707	G/A	-	A/V	306	deleterious	0,04	probably damaging	0,923
rs872935	8:1446587118:144658711	G/A/C	0.449 (A)	L/V	305	deleterious	0,04	probably damaging	0,999
rs367982076	8:1446587148:144658714	C/T	-	A/T	304	tolerated	0,06	probably damaging	0,939
rs147107119	8:1446587168:144658716	A/G	-	V/A	303	deleterious	0	probably damaging	0,999
rs370157447	8:1446587288:144658728	T/G	-	N/T	299	deleterious	0	probably damaging	0,997
rs372583646	8:1446587418:144658741	T/C	-	S/G	295	deleterious	0	probably damaging	1
rs199622906	8:1446588238:144658823	C/A	-	V/L	292	deleterious	0,02	possibly damaging	0,519
rs143378632	8:1446588318:144658831	G/A	-	T/I	289	deleterious	0	possibly damaging	0,799
rs145759182	8:1446588468:144658846	T/C	-	Q/R	284	deleterious	0	benign	0,142
rs267601812	8:1446588598:144658859	G/A	-	P/S	280	deleterious	0	probably damaging	1
rs148205878	8:1446588918:144658891	C/T	-	R/Q	269	deleterious	0,01	benign	0,011
rs374205789	8:1446588958:144658895	C/T	-	E/K	268	deleterious	0	probably damaging	0,988
rs199672137	8:1446589168:144658916	C/A	0.000 (A)	V/L	261	tolerated	0,65	benign	0,001
rs373667786	8:1446589198:144658919	C/G	-	G/R	260	tolerated	0,58	benign	0,005
rs199843319	8:1446589698:144658969	G/A	-	A/V	243	tolerated	1	benign	0,004
rs150754257	8:1446589798:144658979	C/T	-	V/M	240	tolerated	0,27	benign	0,094
rs139128977	8:1446589918:144658991	C/T	-	E/K	236	tolerated	0,89	benign	0,001
rs368314681	8:1446590028:144659002	G/A	-	P/L	232	tolerated	0,24	benign	0,272
rs201161077	8:1446590058:144659005	G/A	-	A/V	231	tolerated	0,32	benign	0,017
rs200176063	8:1446592378:144659237	C/T	0.002 (T)	D/N	227	tolerated	0,33	benign	0,003

rs146015422	8:1446592428:144659242	G/A	-	P/L	225	tolerated	0,52	benign	0,043
rs373219862	8:1446592498:144659249	C/T	-	E/K	223	tolerated	0,11	possibly damaging	0,821
rs139975718	8:1446592548:144659254	C/T	-	G/D	221	tolerated	0,13	probably damaging	0,955
rs144223221	8:1446592918:144659291	C/T	-	G/R	209	deleterious	0	probably damaging	0,999
rs142312560	8:1446593308:144659330	T/G	-	N/H	196	deleterious	0	probably damaging	1
rs111826960	8:1446595418:144659541	G/A	-	R/C	156	deleterious	0	probably damaging	0,991
rs376323296	8:1446598918:144659891	A/C	-	S/A	125	tolerated	0,1	benign	0,009
rs111763816	8:1446599088:144659908	A/G	-	V/A	119	deleterious	0,01	probably damaging	0,998
rs369745388	8:1446599978:144659997	C/T	-	A/T	115	deleterious	0,01	possibly damaging	0,713
rs373884377	8:1446600068:144660006	C/T	-	G/S	112	deleterious	0	probably damaging	1
rs200364051	8:1446600248:144660024	C/T	-	V/M	106	tolerated	0,16	benign	0,153
rs186257137	8:1446600548:144660054	G/A	0.000 (A)	L/F	96	deleterious	0	probably damaging	0,973
rs201407810	8:1446600638:144660063	A/G	0.000 (G)	F/L	93	tolerated	0,06	benign	0,053
rs376515994	8:1446600728:144660072	G/T	-	P/T	90	tolerated	0,23	possibly damaging	0,811
rs200997239	8:1446601068:144660106	C/G	0.001 (G)	Q/H	78	deleterious	0,05	possibly damaging	0,62
rs11990768	8:1446602718:144660271	C/T	-	R/Q	73	tolerated	0,07	benign	0,002
rs35914195	8:1446603188:144660318-14	GG/TA	-	A/V	57	deleterious	0,01	benign	0,08
rs896950	8:1446603198:144660319	G/A	0.014 (A)	A/V	57	deleterious	0,01	benign	0,08

Suplementary Table S5. Predicted impact of the *NAMPT* and *NAPRT1* variants found in our study. Variants were retrieved from 1000 Genomes Browser and scored according to the CADD server (http://cadd.gs.washington.edu/score).

Gene	ID	Chr	Position	Variant type		Cscore	Splice annotation
NAMPT	rs41430346	7	105913155	Non-coding	-	2,069	NA
	rs375379216	7	105913141	Non-coding	-	-	-
	rs112487390	7	105912865	Non-coding	-	11,33	NA
	rs2302559	7	105903904	Silent	Ser301Ser	7,273	NA
	rs144888107	7	105903789	Non-coding	-	4,783	NA
NAPRT1	rs2015562	8	144660257	Non-coding	-	1,111	DONOR
	rs896953	8	144660189	Non-coding	-	0,123	NA
	rs896954	8	144660046	Silent	Ala98Ala	11,6	NA
	rs200364051	8	144660024	Missense	Val106Met	-	-
	rs145565666	8	144659998	Silent	Leu114Leu	13,01	DONOR
	rs2305496	8	144659949	Non-coding	-	4,955	NA
	-	8	144659838	Silent	Val142Val	-	-
	rs12678314	8	144659608	Non-coding	-	5,774	NA
	rs744650	8	144658730	Silent	Pro298Pro	3,054	ACCEPTOR
	rs872935	8	144658711	Silent	Leu305Leu	1,158	NA
	rs35975875	8	144658630	Missense	Arg332Cys	20,9	NA
	rs114291348	8	144658505	Non-coding	-	3,723	NA
	-	8	144658501	Non-coding	-	-	-
	rs896955	8	144658370	Non-coding	-	6,296	NA
	-	8	144657972	Non-coding	-	-	-
	rs2290417	8	144657269	Non-coding	-	14,31	ACCEPTOR
	rs77951814	8	144657152	Non-coding	-	16,73	DONOR
